# Preclinical and Clinical Effects of Mistletoe against Breast Cancer

**DOI:** 10.1155/2014/785479

**Published:** 2014-07-20

**Authors:** Mohsen Marvibaigi, Eko Supriyanto, Neda Amini, Fadzilah Adibah Abdul Majid, Saravana Kumar Jaganathan

**Affiliations:** ^1^IJN-UTM Cardiovascular Engineering Center, Faculty of Biosciences and Medical Engineering, Universiti Teknologi Malaysia, 81310 Skudai, Johor, Malaysia; ^2^Bioprocess Engineering Department, Faculty of Chemical Engineering, Universiti Teknologi Malaysia, 81310 Skudai, Johor, Malaysia

## Abstract

Breast cancer is among the most frequent types of cancer in women worldwide. Current conventional treatment options are accompanied by side effects. Mistletoe is amongst the important herbal medicines traditionally used as complementary remedies. An increasing number of studies have reported anticancer activity of mistletoe extracts on breast cancer cells and animal models. Some recent evidence suggests that cytotoxic activity of mistletoe may be mediated through different mechanisms. These findings provide a good base for clinical trials. Various studies on mistletoe therapy for breast cancer patients revealed similar findings concerning possible benefits on survival time, health-related quality of life (HRQoL), remission rate, and alleviating adverse reactions to conventional therapy. This review provides an overview of the recent findings on preclinical experiments and clinical trials of mistletoe for its cytotoxic and antitumor activity and its effect on HRQoL in breast cancer patients. Moreover, studies investigating molecular and cellular mechanisms underlying antitumor activity of mistletoe are discussed in this paper. The analyzed trials provided evidence that there might be a combination of pharmacological and motivational aspects mediated by the mistletoe extract application which may contribute to the clinical benefit and positive outcome such as improved HRQoL and self-regulation in breast cancer patients.

## 1. Introduction

Breast cancer is the most common type of nonskin malignancy among women worldwide. It has been reported that the incidence and mortality of breast cancer have increased during the last two decades [1–3]. Based on 2006–2010 statistics, the number of deaths in the United States was 22.6 per 100,000 women per year. It is predicted that estimated 232,670 new cases of breast cancer and 40,000 breast cancer-related deaths will occur among women in 2014 worldwide [4]. It has been shown that the progression of primary and secondary tumors into unlimited proliferation has a vital role in pathogenesis of breast cancer. Breast cancer progression varies significantly in different cases due to the fact that tumor malignancies differ in molecular characteristics, prognosis, and invasiveness [5]. Early diagnosis of breast cancer using current methods of early detection allows for more effective treatment of breast cancer. Mammography as the most reliable option remains the first screening test for early detection of breast cancer [6, 7]. Although many treatment methods are currently established including surgery, radiotherapy, biological therapy, hormone therapy, and chemotherapy, these therapies are less effective and recurrence is still an issue in breast cancer patients due to side effects, toxicity of drugs in normal cells, and aggressive behaviour of the tumors [8–11]. In spite of many improvements in the use of hormonal and adjuvant cytotoxic therapies for breast cancer patients, there has been no considerable reduction in the mortality of breast cancer today [5]. The treatment success rate remains unsatisfactory, thus it is essential to find other adjuvant methods for breast cancer therapy. Complementary and alternative medicine (CAM) as one of the major aspects of cancer therapy has been developed in the last few years in order to alleviate the side effects of drugs and relieve the pain in breast cancer patients [12]. Several studies have been carried out to observe the effects of CAM in breast cancer patients. CAM may be valuable for optimizing the conventional therapy [13, 14]. Medicinal plants can be used in combination with conventional medicine as a supportive therapy to improve health-related quality of life (HRQoL). It has been shown that the use of some types of CAM in breast cancer patients has dramatically increased and is gaining in popularity [15–17].

## 2. Mistletoe

As an anthroposophical medicine, mistletoe is one of the most important herbal drugs and is potentially effective against cancer [18]. Using mistletoe extract for cancer therapy especially breast cancer is recommended due to its minimal side effects and the fact that these side effects are not life threatening. A few cases of allergic reactions have been reported [19]. Rudolf Steiner, founder of anthroposophy medical method, introduced mistletoe extract into oncology as an unconventional means of therapy for cancer in 1920 [20].

### 2.1. Mistletoe Active Compounds

**Figure 1:**
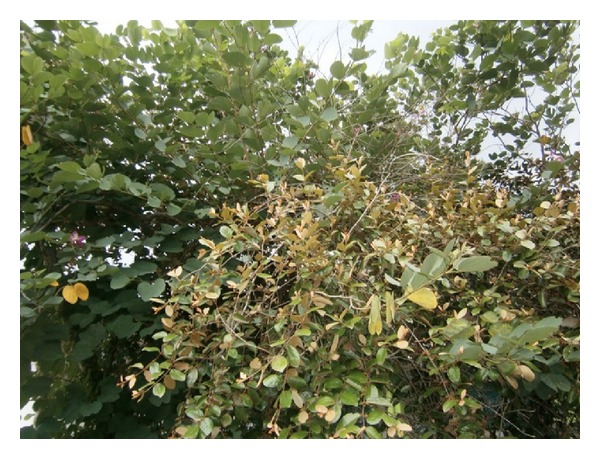
Mistletoe on host tree.

**Figure 2:**
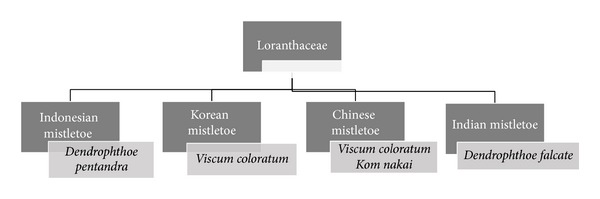
Location of growth and scientific names of mistletoes belonging to Loranthaceae family.

**Figure 3:**
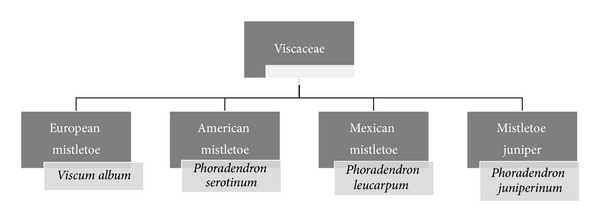
Location of growth and scientific names of mistletoes belonging to Viscaceae family.

Mistletoe is an evergreen semiparasitic shrub (Figure 1) from the Viscaceae (Loranthaceae) family with various types which can grow on the branches of different deciduous trees like tea, apple, pine, mango, lime, larch, pear, oak, and some other plants [21, 22]. Worldwide, approximately 1500 species of mistletoe have been identified [22]. As depicted in Figures 2 and 3 various kinds of mistletoe have been selected by researchers to study their anticancer effect. Mistletoe contains different types of biological active compounds such as carbohydrates, fats, amino acids, oligosaccharides, polysaccharides, enzyme, flavonoid, glycoprotein (lectin MLT), polypeptide (viscotoxin), vesicles, and triterpene acids [23–29]. The chemical composition of mistletoe varies depending on the techniques of extract preparation, season and time of harvesting, commercial producer, stage of growth of the plant, location, and species of host tree [30, 31]. Lectins (ML-I, ML-II, and ML-III) are the main constituents of mistletoe which are responsible for its antitumor and immunomodulatory effects [32–34]. Today, most researchers have focused their studies on mistletoe lectins, particularly mistletoe lectin I (ML-I). The mistletoe lectins are glycoproteins belonging to the ribosome inactivating proteins family type II. They consist of an A-chain of 254 amino acids (MW 28.480 kDa) and a B-chain of 264 amino acids (MW 28.960 kDa) connected to each other by a disulphide bridge [35].

The anticancer effect of ML-I depends on activities exerted by both the A-chain and the B-chain. Carbohydrates are bound to the B-chain which mediates the cellular uptake of the hololectin. Cytotoxic A-chain inhibits the elongation step of protein biosynthesis by catalysing hydrolysis of the N-glycosidic bond at adenine-4324 in the 28S RNA of the 60S subunit of ribosomes, resulting in apoptosis or necrosis cell death [36, 37]. The immunomodulatory activity of mistletoe lectin is attributed to B-chain manifested by enhancing the secretion of cytokines and the activity of natural killer cells [38, 39]. Significant increases of natural killer and helper T-cells that are generally believed to be involved in antitumor activity have been observed in 20 mammary carcinoma patients who received ML-1 by subcutaneous injections [40]. In a recent study by Maletzki et al. in 2013 [41], it has been stated that there is high resemblance between the 3D structure of mistletoe lectin and the shiga toxin from* Shigella dysenteriae*, which represents the bacterial origin of this protein. This finding justifies the significant immunogenicity of mistletoe lectin. Moreover, the researchers suggested combining mistletoe lectin with other forms of recognition receptor ligand substances to enhance the immune stimulatory effect.

The antimutagenic potential of mistletoe lectin (*Viscum album* L. var.* coloratum* agglutinin) was assessed by Hong and Lyu in 2012 [42]. Their results showed moderate to negligible protective ability of VCA to various mutagens in* S. typhimurium* TA100 and TA98 strains. The spectroscopic study of Bogoeva et al. in 2013 [43], revealed a novel property of ML-I to bind cytokinins with high affinity. Their findings suggested possible relation of this protein to the group of phytohormone-(cytokinin)-binding proteins. Stauder and Kreuser reviewed the preclinical studies and clinical trials in terms of mistletoe lectins (ML-I) in cancer populations. They reported immunostimulating and cytotoxic effects of mistletoe extract in numerous preclinical experiments. Moreover, they suggested that more high quality, well planned randomized clinical trials are needed to verify the effect of mistletoe ML I on the improvement of HRQoL in cancer patients [44]. Apart from the mistletoe lectins, viscotoxins are the most important substances of mistletoe. Viscotoxins are polypeptides consisting of 46 amino acids and three disulphide bridges. It is well known that they have immunogenic effects [45, 46]. In addition to viscotoxin and lectins, mistletoe contains other types of peptides known as Kuttan's peptides. It shows some immunostimulatory and cytotoxic effects against tumor cells [47].* Viscum album* (VA) is the European type of mistletoe which has been widely studied by researchers. Numerous preparations of this plant from different host trees like apple, pine, oak, and others have been used. Many mistletoe extract preparations are commercially available including Isorel, Cefaleksin, Lektinol, Eurixor, Iscador, Helixor, Iscucin, and Abnobaviscum which are marketed as injectable prescriptions and can then be used as adjuvant therapy for the treatment of cancer [48]. While numerous studies concerning* V. album* have been reported, few investigation of* V. cruciatum* have been carried out. As recommended by Lev et al. in 2011 [49], targeting research on this plant would be beneficial in cancer care.

## 3. Preclinical Studies

### 3.1. *In Vitro* Studies

Several preclinical studies and* in vitro* experiments have investigated the anticancer effect of mistletoe extract and its active compounds in breast cancer and predominantly reported their antitumor and cytotoxic effects in cancer cell lines (Table 1). They all agree on this point that mistletoe extracts exhibit substantial cytotoxic effects* in vitro* and none of the studies reported growth stimulation and proliferation of tumor cell lines [50–62].* Viscum liquidambaricolum* and* Viscum coloratum* are known as Korean mistletoes which belong to Loranthaceae family and have been used as a traditional medicine in Korea. It has been shown that isolated triterpenoids and triterpenoid saponin of* Viscum liquidambaricolum* represent cytotoxic activity towards MCF7 cell line [50]. The cytotoxic activity of flavonoid compounds of isolated pachypodol and ombuine identified from Chinese mistletoe (*Viscum coloratum* Kom, Nakai) against MCF7 cell line has been demonstrated* in vitro* using MTT assay [51]. African researchers studied the antibreast cancer activity of Cameroonian mistletoe using MB-MDA 435 cell line. Their findings demonstrated significant reduction of breast cancer cell viability after mistletoe treatment [52]. In another study, Iranian researchers investigated the cytotoxic effect of Iranian mistletoe on breast cancer cell line MCF7 using MTT assay. They found the mistletoe plants growing on hornbeam tree (*Carpinus betulus*) effective against breast cancer cell line MCF7. Mistletoe plant juice or hydroalcoholic extract showed the highest cytotoxic activity on cancer cell line [53].

The cytotoxic activity of* Dendrophthoe falcata* known as Indian mistletoe on MCF7 cell line using MTT, SRB, and brine shrimp lethality bioassays was studied by Indian researchers. Cell proliferation was significantly inhibited after treatment with ethanolic and aqueous extracts of* D. falcata*. Aqueous extract represented higher cytotoxic activity [54]. In experiments using American mistletoe, investigators isolated four novel small proteins, phoratoxins or ligatoxins from acetic acid extract of California mistletoe,* Phoradendron tomentosum* growing on* Populus fremontii* S. Watsonby reversed phase chromatography. In addition, cytotoxic properties of* P. tomentosum* were examined using an* in vitro* fluorometric microculture cytotoxic assay. The isolated proteins exhibited cytotoxicity towards various cell lines. Tumor cells treated with phoratoxin C exerted higher activity. The activity of phoratoxin C was evaluated on the primary culture of human tumor cell from patients. Phoratoxin C showed remarkable toxicity against the solid tumor from breast carcinoma [55]. In a further study, the anticancer effects of other types of American mistletoe,* Phoradendron serotinum*, on proliferation and apoptosis of human breast cancer cell line were investigated by Mexican scientists. They reported that extract of* P. serotinum* exerted cytotoxic activity on MCF7 cell line [56].

Most of the studies focusing on mistletoe as a candidate complementary drug for cancer therapy have been performed by European scientists, especially researchers in Germany who carried out their preclinical and clinical experiments using different commercial and standardized products of* V. album*. The cytotoxic effect of mistletoe extract with a defined content of bioactive mistletoe lectin on various human tumor cell lines and xenografts has been reported by Burger et al. [63]. In his study, the antiproliferative activity was compared to classical anticancer agents and high cytotoxic activities were observed in human cancer cells. None of the tested tumor cell lines showed increased rate of proliferation. Antitumor profiles of mistletoe were found to be similar to doxorubicin which is an anticancer agent. Further* in vitro* studies conducted by Burger et al. in 2003 [64] recorded no stimulation of MCF-7 cell proliferation using mistletoe extract with a defined content of bioactive mistletoe lectin. The effects of different Helixor mistletoe extracts including Helixor P, Helixor A, and Helixor M on proliferation and growth stimulation of 38 human cancer cell lines have been studied by Kelter et al. [57]. They used mistletoe lectin I along with adriamycin and interleukin 6 (IL6) as reference compounds and carried out their studies using various human breast cancer cell lines. They found that different Helixor extracts have cytotoxic activity on tumor cells and also suggested that no growth stimulation of human cancer cell lines occurred. A study was performed by Maier and Fiebig in 2002 to investigate the antitumor and antimetastatic activity of aqueous mistletoe extract in a panel of 16 human tumor cell lines [58]. They concluded from their work that all Iscador mistletoe extracts used in their* in vitro* experiment showed no tumor proliferation and growth stimulation in cancer cells. They also observed that Iscador M spezial and Iscador QU spezial that contain high amounts of lectin showed anticancer activity in the mammary cancer cell line MAXF401 NL.

Using* in vitro* approach, Knöpfl-Sidler et al. in 2005 [59] showed that treatment of MFM-223, KPL-1 cell lines with Iscador M, Iscador Q, and* Abnobaviscum Fraxini-2* resulted in the inhibition of proliferation of cancer cells.* Abnobaviscum Fraxini-2* exerted strong growth inhibitory effects against MFM-223. In 2006, Beuth et al. [60] studied the human ductal breast carcinoma cell line BT474 and proved the direct antitumor efficacy of Helixor M and Helixor A using a calorimetric* in vitro* assay. The tumor necrosis factor alfa (TNF*α*) was used as a reference compound for* in vitro* cytotoxic tests. Their work demonstrated a dose-dependent antitumor activity of all tested mistletoe extracts against BT474 cells. Standardized mistletoe significantly represented more cytotoxicity compared to TNF*α*. In addition, dramatic reduction of tumor weight as well as a decreased cell proliferation rate was reported in their* in vivo* study. In further studies by Hugo et al. in 2007 [61], HER2 cell line were used as model cell lines for malignant EGFR/c-erb B-2 double positive breast cancer for anticancer analysis. Their findings showed that Iscador M, which contains high amount of mistletoe lectin, can inhibit the epidermal growth factor proliferation of human breast cancer cell line. Moreover, their results recommended* V. Album* extracts as an antitumor drug, especially for breast cancer therapy. The cytotoxic activity of different* V. album* preparations on breast cancer cell lines using calorimetric assay was studied by Eggenschwiler et al. in 2007 [62]. They compared the anticancer effects of VAP-M, VAP-Qu, VAP-P, VAP-A, and purified mistletoe lectin. It was shown in their work that each of the VA preparations caused inhibition of cell lines proliferation but that the extent of growth inhibition differed according to the types of preparations and cell lines. Their results provide direct evidence of antitumor properties of different Iscador preparations against various breast cancer cell lines. They also found new possibilities to investigate the therapeutic potential of VAP-A in breast cancer therapy caused by the existence of cytotoxic substances other than mistletoe lectin. From the preclinical investigations discussed above, it is evident that various mistletoe extracts from different origins are capable of inducing apoptosis and cell death in human breast cancer cell lines. Most of the* in vitro* and preclinical studies have revealed that mistletoe might be a good candidate for development of an anticancer drug [50–62].

### 3.2. *In Vivo* Studies

In addition to preclinical* in vitro* investigations, many* in vivo* experiments using different animal models have been conducted by researchers. The cytotoxic and antitumor activity of mistletoe, especially the European mistletoe, against a variety of rats and mice has been demonstrated in most of the investigations. Reduction in the tumor size and growth has been reported in some of the studies [60, 67].* V. album* extract has been applied by subcutaneous injection in most reported studies on European mistletoe; however, other administration routes such as oral, intramuscular, intrapleural, intraperitoneal, and intratumoral on relevant site have been described in the experiments [60, 66, 67]. Drees et al. in 1996 [67] reported the reduction of cell proliferation in MAXF 449, sc/Nude mice using Abnobaviscum M. In 2006, Beuth et al. [60] demonstrated the dose-dependent antitumor activity of Helixor using a BALB/c-mouse/BT474 ductal breast carcinoma model. In their* in vivo* experiment, ME-A and ME-M were applied to the breast carcinoma model intratumorally. As compared to the control group, tumor treated with Iscador showed a noticeable reduction in cell growth and proliferation with decrease in tumor weight as well as increase in cell apoptosis. In contrast, no significant antitumor and cytotoxic activity was observed with Iscador subcutaneously injected to the three chemosensitive transplantation tumor models involving 214 rats and 93 mice [66].* In vivo* investigations of the ability of mistletoe extract to improve tumor survival induce apoptosis and necrosis and inhibit cancer cell proliferation in animal models that have yielded inconsistent results. However, some research works showed decrease in the rate of cell proliferation and improvement in tumor survival.

## 4. Clinical Studies

Most of the breast cancer patients especially women in Europe using adjuvant medicine in addition to chemotherapy, nevertheless, show evidence of its efficacy on HRQoL and survival of these patients is still a controversial topic of discussion. The majority of researchers employed a combination of* V. album* extract therapy and conventional therapy in the treatment of breast cancer patients after surgery and radiotherapy. Improvement in HRQoL, positive influence in remission rate, and reduction of side effects were observed in most studies carried out on patients who received mistletoe extract in addition to chemotherapy [68–75]. Life satisfaction (LS) and HRQoL are two main important factors which are needed to be taken into consideration for treatment of breast cancer patients. Systematic reviews and clinical reports of various single arm cohort and case series studies on mistletoe therapy have appeared in the medical literature since 1970. Overall, there is evidence that mistletoe therapy may enhance the survival rate, improve HRQoL, and diminish the side effects of chemotherapy in breast cancer patients as concluded in many systematic reviews [76–87]. Researchers mainly tested the effectiveness of mistletoe on HRQoL, survival time, tumor behaviour, and side effects of chemotherapy and radiotherapy in women with breast cancer. In contrast, application of mistletoe extract additional to conventional therapy shows weak and inconsistent results on survival time of the other types of cancer patients. Tables 2 and 3 present a summary of the clinical trials and systematic reviews on the benefits of mistletoe therapy in breast cancer patients performed during the last decade. A multicentre three-arm study on 643 women with operated breast cancer was carried out by Gutsch et al. in 1988 [88]. The patients were divided into 3 therapeutic groups. The follow-up group involved 274 patients, while 192 female patients were treated with Helixor and the remaining 177 patients received chemotherapy. Compared to the untreated control group, better survival rates were observed in the two treatment arms. Some weak evidence provided by the 2-arm randomized clinical study of Heiny in 1991 [89], with 46 advanced breast cancer patients showed that Eurixor may have positive effects on HRQoL during palliative chemotherapy. In another small 2-arm randomized clinical trial, Borrelli in 2001 [90] assessed HRQoL and tumor response in women with metastatic breast cancer. Overall, 30 breast cancer patients were randomly assigned to receive either mistletoe extract or placebo. Mistletoe extracts were administered subcutaneously. The researchers assessed HRQoL with Spitzer's QLI. The author stated special benefits in the measurement of HRQoL as well as an influence in tumor response in breast cancer patients treated with mistletoe extract. In 2001, Grossarth-Maticek et al. [91] performed a multicancer prospective long-term epidemiological cohort study on the efficacy and effectiveness of Iscador on survival times including 10226 patients with different types of malignancy. The survival times of the patient group treated with Iscador were superior to those of the control group. The authors found sufficient evidence to recommend the use of Iscador for treatment of cancer in breast cancer patients with or without axillary metastases. In 2002, Kröz et al. [92] reported some benefits of mistletoe therapy in survival and tumor reduction in women with metastasized breast cancer. They applied combined intra-, peritumoral, and subcutaneous* abnoba-Viscum(r)* with concomitant pamitron acid to breast cancer patients. Improvement in HRQoL was observed in 80-year-old women who received the mistletoe therapy subcutaneously.

A multicentric retrolective cohort study was designed by Schumacher et al. in 2003 [93], to investigate the effect of lectin-standardized mistletoe extract on HRQoL in 1248 breast cancer patients. 689 patients who were subjected to final analysis received standard conventional therapy and were divided into two groups: a therapy group including 219 patients with additional complementary therapy and a control group with 470 patients who received no additional complementary treatment. An assessment of relapse-free treatment and HRQoL was carried out after follow-up times of 284 and 285 days for the treatment and control groups, respectively. Data analysis of the patients in therapy groups showed improvement in HRQoL, prolongation of relapse intervals, and reduction of side effects caused by tumor destructive therapies.

Enhancement in survival time and improvement in HRQoL were assessed in pharmacoepidemiological, retrolective cohort study with Iscador by Bock et al. in 2004 [68]. Out of a total of 1442 patients with primary nonmetastasized breast carcinoma, 710 patients were given mistletoe treatment and 732 patients were considered as the control group. Satisfactory HRQoL and significantly less adverse reactions to the adjuvant oncology treatments were observed in patients who received mistletoe therapy. An approval randomized prospective clinical trial was performed by Piao et al. in 2004 [69], for Chinese people. A total of 68 breast cancer patients provided with standard conventional therapies were enrolled into this study. Helixor A was administered subcutaneously to patients of the treatment group, while the control group received Lentinan intramuscularly. HRQoL was measured by approval questionnaire including traditional Chinese medicine (TCM) and functional living index (FLIC). Analysis of the results demonstrated that mistletoe complementary therapy could improve HRQoL and reduce standard therapy adverse reactions. Another randomized, double blind, multicentre, and placebo controlled clinical trial was done by Semiglasov et al. in 2004 [94]. A total of 272 women with operable breast cancer were selected from 9 centres in Russia, Bulgaria, and Ukraine. All breast cancer patients receiving chemotherapy (cyclophosphamide-methotrexate-fluorouracil) were randomized to placebo or standardised mistletoe PS76A2 groups. The researchers of this study compared different doses of mistletoe extract preparation with a placebo treatment. Their results revealed that PS76A2 is effective in HRQoL improvement in breast cancer patients receiving adjuvant chemotherapy.

Further investigation was done by Semiglazov et al. in 2006 [70]. They carried out a prospective, multicentre, and double blind study with similar trial protocol in order to confirm their own previous findings with PS76A2. The outcome showed that PS76A2 led to significant improvement in HRQoL in female subjects with breast cancer during chemotherapy and follow-up without chemotherapy. As mentioned above, Semiglasov obtained similar results from clinical studies they carried out in 2004 and 2006. On the other hand, results obtained from a double blind study of Auerbach et al. in 2005 [95], on early breast cancer patients receiving radiochemotherapy showed no significant effect of Helixor A adjuvant therapy on HRQoL. In a matched pair design clinical trial including 84 pairs of nonrandomized and 38 pairs of randomized breast cancer patients in 2006, the effect of Iscador on self-regulation and survival time was studied by Grossarth-Maticek and Ziegler [96]. A significant benefit of long-term Iscador therapy with respect to mean survival time and improvement in the self-regulation was reported in patients who received Iscador. In a multicentric and comparative clinical trial by Beuth et al. in 2008 [71], the study population was drawn from 741 women with primary breast cancer who participated in an epidemiological cohort study on the influence of complementary Helixor treatment on HRQoL. Among the study group, 167 patients were treated with Helixor and other 514 patients remained as the control group. Safety and efficacy were set as the endpoints in this clinical investigation. Significant reduction in the persistent symptoms of disease and treatment, as well as improvement in HRQoL, was seen during aftercare in patients who received mistletoe extract. Büssing et al. in 2008 [72] performed a clinical study in which Iscador M Spezial was administered intravenously to 65 breast cancer patients. The tolerability of intravenously applied* V. album* was good at concentrations between 1 and 8 mg. Analysis of all patients' results in the treatment group showed a significant positive impact on chemotherapy side effects such as nausea, constipation, stomatitis, and pain. Nevertheless, no significant effect on HRQoL improvement was observed in treatment group.

Another double-arm nonrandomized clinical trial involving 33 breast cancer patients was carried out by Loewe-Mesch et al. in 2008 [97]. The group of patients who received mistletoe Iscador M spezial therapy showed fewer side effects to the conventional therapy. Another positive result with mistletoe therapy was reported in female patients with early-stage breast cancer by Tröger et al. in 2009 [73]. In this open label and randomized pilot clinical trial, patients were stratified into 3 groups that underwent CAF chemotherapy. Patients of both strata were then subjected to either Iscador M Spezial extract or other preparations of mistletoe. Patients of third strata received no additional therapy as a control group. HRQoL and fatigue as the outcomes were assessed by EORTC-QLQ-C30. The research findings demonstrated a tendency to reduction of neutropenia and improvement of HRQoL in breast cancer patients treated with IMS additional to CAF. According to the author's comment, limited sample size was the drawback of this study and further studies with large sample size were needed to prove the results.

Cancer related fatigue (CRF) is the most common unrelieved symptom and side effect of chemotherapy, radiotherapy, and biotherapy which impairs HRQoL of patients significantly and extensively more than other symptoms. The impact of Iscador M on CRF was studied on a 36-year-old patient with a 10-year history of recurrent breast cancer. Dose-dependent benefit in decreasing fatigue was observed during two and half years of treatment with mistletoe extracts [98]. A report of an observational multicentre study was published by Eisenbraun et al. in 2011 [74], including 270 patients with breast cancer of stages I–III. The study investigated the effectiveness of abnoba-Viscum M on HRQoL. The researchers measured effectiveness and tolerability of mistletoe therapy additional to chemotherapy under the conditions of daily practice. This noninterventional clinical trial showed improvement in HRQoL. In a further study, Tröger in 2011 [99] investigated the connection between neutropenia and HRQoL in 95 breast cancer patients. A trend toward neutropenia reduction and better HRQoL was observed in the group who received Iscador M spezial in addition to chemotherapy. These obtained results were similar to their previous findings mentioned earlier [73]. Werner et al. in 2011 [100] conducted a multicenter, controlled, and retrolective study enrolling 3376 patients diagnosed with various types of cancer including breast cancer. They evaluated the therapeutic efficacy and tolerance of Iscador P, Qut, and M applied in addition to palliative conventional therapy in patients. All types of Iscador were found to be effective. Significant reduction in tumor related symptoms and prolonged overall survival was observed in the group receiving mistletoe extracts as a supportive therapy. An exploratory cohort clinical investigation was conducted by Brandenberger et al. in 2012 [101], where the efficacy of Isorel, Iscador, and abnoba-Viscum on patients with various types of cancer including 4 breast cancer cases were studied. Pooled analysis of EORTC QLQ-C30 questionnaire and interview content suggested that three-month adjuvant* V. album* therapy is associated with a decrease in CRF and better HRQoL in cancer patients. In a recent study, a prospective clinical five-year follow-up trial has been designed to investigate the influence of mistletoe therapy in combination with chemotherapy on the relapse and overall survival time. An examination of relapse and survival time was carried out after a follow-up time of five years. Analysis of the results from all patients with early breast cancer in treatment group who received Iscador M spezial showed an increase in HRQoL of patients. There was no report on negative impacts of adjuvant mistletoe therapy on chemotherapy effectiveness [75]. In a recent randomized pilot study, Tröger et al. (2014) [102] have obtained findings consistent with those found by Piao et al. [69], wherein breast cancer patients who received Helixor A in addition to adjuvant CAF chemotherapy showed remarkable improvements of HRQoL.

In spite of differences in the exclusion and inclusion criteria, quality rating, and mistletoe preparations, numerous clinical trials on mistletoe therapy for breast cancer patients show similar findings concerning possible benefits on the survival time, HRQoL, and reduction of standard therapies adverse reactions (Tables 2 and 3). Most of the clinical studies on the efficacy of mistletoe extract on HRQoL and survival time had been conducted in Germany and some in Switzerland, Russia, Serbia, and Ukraine.

### 4.1. Limitations of the Clinical Trials

Registration of small numbers of breast cancer patients is the main limitation of clinical trials [73]. Lack of control group is the other weakness of such trials. Blind pharmaceutical studies may experience confounding issues which limit these trials. However, findings obtained from Semiglasov's studies [70, 94] have verified that blinding may not necessarily have had relevant effects on the results of mistletoe studies.

## 5. Mechanisms Involved in the Antitumor Activity of Mistletoe against Breast Cancer Cells

### 5.1. Apoptosis Induction

Some recent evidence obtained from experiments in cell cultures and animal models suggests that cytotoxic and antitumor activities of mistletoe may be mediated through different mechanisms including induction of apoptosis and necrosis, inhibition of cell cycle progression, activation of specific or nonspecific immune system, and release of beta-endorphin into plasma. However, the precise cellular and molecular mechanisms and the signal transduction pathways underlying the issue of how mistletoe extracts induced apoptosis have not been completely revealed.

The effects of cytotoxic lectins isolated from Korean mistletoe (KML-C) on proliferation and apoptosis of various human and murine tumor cells were investigated by Yoon and fellow researchers in 1999 [103]. A comparative study on biological and chemical properties of KML-C and lectin from European mistletoe (EML-1) was carried out. They observed that the cytotoxic activity of KML-C against different tumor cells was stronger than that of EML-1. The induction of apoptosis characterized by morphological changes of apoptotic nuclei and DNA fragmentation were reported in tumor cells incubated with KML-C. Their findings showed that addition of Zn^2+^ significantly blocks apoptosis inducing activity of KML-C in a dose-dependent pattern. These results suggest antitumor activity of KML-C resulting from Ca^2+^ Mg^2+^ dependent endonucleases and a consequent apoptosis induction. In 2006, Harmsma and coworkers [104] examined the effects of different concentrations of Iscador M and Iscador Q on nine various human tumor cell lines with regard to induction of apoptosis and mechanism of action. The data showed that Iscador caused early cell cycle regression followed by induction of apoptosis in a dose-dependent manner in breast cancer cell line. Moreover, results represented that Iscador Q induces apoptosis via mitochondrial pathway, while Iscador M induces apoptosis by activating death receptor pathway.

In experiments using Iscador Q, Iscador M, and Iscador P, Ramaekers et al. in 2007 [65] indicated that different Iscador preparations can induce cell cycle inhibition and tumor cell regression. They observed concentration-dependent inhibition in cell cycle machinery, particularly complete inhibition of S-phase progression in MCF7 breast cancer cell line. Cytostatic effect occurred in a dose-dependent manner. In addition, they investigated the mechanism of antimetastatic and anticancer activity of Iscador at molecular and cellular level. In order to detect apoptotic pathways involved in Iscador induced programmed cell death, the cells were stained for several proapoptotic related components. Immunostaining for proapoptotic specific proteins in Iscador Q treated cells, indicated involvement of the mitochondrial route, whereas Iscador M treated cells revealed involvement of both mitochondrial and death receptor pathways. Furthermore, Iscador P was shown to be less effective in the induction of apoptosis and more effective in the induction of necrosis due probably to the presence of viscotoxins. Mistletoe extract seems to induce apoptosis via activation of death pathways, possibly through activation of caspase 8 and subsequently activation of other cascade of caspases in apoptotic process and by loss of mitochondrial potential which, in turn, release cytochrome C into cytosol and activate caspase 9.

### 5.2. Immune System Modulation

Numerous scientific studies have confirmed that mistletoe extracts are capable of boosting the immune system which can enhance tumor destruction or shrinkage [105–119]. Several research studies have been conducted in order to acquire a better insight into the mechanism of action of mistletoe extracts on the immune system as it remains elusive. Controlled bicentric clinical trial using 105 breast cancer patients was carried out by Büssing in 2006 [105]. Minimized immune suppression was observed in patients who received Iscador M spezial intravenously. In an investigation of immunomodulatory effects of Iscador, Hajto in 1986 [106] evaluated various immunological parameters in the peripheral blood of breast cancer patients. Six hours after a single intravenous application of VA-E, a significant enhancement of granulocytes function and number were recorded. Within 24 hours, remarkable increase in antibody-dependent cell-mediated cytotoxicity (ADCC) and natural killer cell activities as well as an increase in the uptake of [3H]-thymidine in the DNA of mitogen-stimulated lymphocytes were observed. Beuth et al. in 1992 [107] investigated the immunomodulatory capacity of galactoside-specific lectin from mistletoe in 10 breast cancer patients who had been provided with conventional therapy. They analyzed the behavior of various lymphocyte subsets, NK cells, and the level of expression of activation marker after subcutaneous administration of the optimal doses of ML-1 in patients. Subcutaneous injection of ML-1 resulted in high pronounced release of lymphocyte subsets in the blood of many patients. Enhanced counts of pan T cells, NK cells, and T helper cells involved in antitumor immunity were reported in most of the patients. Furthermore, using* in vitro* assay, it was revealed that exposure of human lymphocyte to various concentrations of ML-1 induced an enhanced expression of HLA-DQ antigens and IL-2 receptors. Further studies have been carried out by Beuth et al. [108, 109], examining the immunomodulatory activity of ML-1 in patients with mammary carcinoma. An increased concentration of acute phase proteins and of complement factor C3 and enhanced expression of IL2 receptors on lymphatic cells were observed during immunotherapy of cancer patients with different concentrations of ML-1. It has been established that there is a correlation between HRQoL and responses of the cellular parameters of immune system in cancer patients. Heiny and Beuth in 1994 [110] demonstrated significant correlation between improved HRQoL and the increased level of plasma beta-endorphin in breast cancer patients. Regular subcutaneous administration of ML-1 in 36 breast cancer patients significantly enhanced the plasma level of beta-endorphin and the* in vitro* cytokine release. Heiny et al. in 1998 [111] studied the correlation between cellular parameters of immune system and neuroendocrine system in breast cancer patients. Increased activity of peripheral blood natural killer (NK) cells and T-lymphocytes as well as enhanced *β*-endorphin plasma levels were reported after mistletoe lectin therapy. The mistletoe lectins obtained from Korean or European mistletoe were investigated for their modulation of tumor cell sensitivity toward TNF*α* by Pae et al. in 2000 [112]. They found that mistletoe lectin increases MCF-7 cell sensitivity to TNF*α* induced apoptosis. Protein synthesis inhibition is perhaps the mechanism by which lectins amplify the effect of TNF*α*. In a pilot study in 2000, Kovacs [113] demonstrated that* V. album* extract as an immunomodulator could result in improved cell mediated immunity. The study was undertaken to assess the effect of VA therapy on the serum level and cytokines production in peripheral blood mononuclear cells in women with breast cancer. Serum level measurement of immunological parameters revealed that patients who received VA therapy showed a significant increase in Th1 cytokines like IL-2 and IFN gamma. In addition, treatment with VA leads to an enhancement in the serum level of IL-12. The level of IL-4 and the number of NK cells and T-inducer cells were not altered during subcutaneous injection of VAE.

The effects of a single perioperative intravenous application of a viscotoxin containing VA-E on granulocytes activity was studied by Büssing et al. in 2005 [114]. A total 105 breast cancer patients were enrolled in their prospective, bicentric, and nonrandomized clinical trials. The patients were divided into two groups, a treatment group receiving a perioperative infusion of Iscador M spezial including 53 patients and a control group with 52 patients. The research findings demonstrated that Iscador M is able to prevent suppression of granulocyte burst activity triggered by surgical stress in breast cancer patients. In addition, the level of C-reactive proteins was not altered in the treatment group. In 2007, Büssing et al. [115] hypothesized that rapid escalation of high VA-E concentration may impair the function of competence of T lymphocytes in cancer patients. The course of T-cell function was stable in 36 breast cancer patients who received moderate mean concentrations of VA-E, while other patients showed a decline in the stimulated T-cell function. A further observational study was undertaken by Büssing et al. in 2008 [116], designed like their previous trial. They evaluated the HRQoL and course of peripheral lymphocyte subset in cancer patients. They concluded from their work that the induction of moderate local reactions can be associated with better HRQoL and T-cell function. In a study in 2006, Heinzerling et al. [117] investigated the immunological effects of therapy course with Iscador Qu spezial and evaluated the mechanism responsible for clinical observations of side effects disappearance in a group of cancer patients. They indicated that mistletoe therapy activated innate immune via CD14 which is mediated by the activation of monocytes. Furthermore, long-term Iscador therapy induced a Th1 response to mistletoe. The specific Th1 response was associated with strong bystander activation resulting in the induction of memory T-cell that might mediate* in vivo* anti-infectious and anticancer activity. In a 4-case series study in 2009, Gardin [118] conducted a small trial on the effectiveness of Quercus therapy on immune stimulation for cancer patients who had immune impairment including a 44-year-old woman with breast cancer. Improvements in many humoral and cellular parameters were observed in patients who received 20 mg VA extract by subcutaneous administration twice a week. The results confirmed that VA has the ability to improve humoral and cellular immune responses in cancer patients. Son et al. in 2010 [119] provided evidence that Helixor exhibited immunostimulatory effects in breast cancer patients. They examined the immunological effects of VAE after surgery followed by standard conventional therapy in 20 patients with stage *Ι* or *Ι* *Ι* primary breast cancer. The test group received VAE by subcutaneous injection. Measurements of various cytokines were performed using ELISA system. Analysis of the results showed that concentration of IL-6 and IFN-gamma in peripheral blood remarkably increased in the test group after VAE therapy. Hagens et al. in 2011 [120] investigated the influence of Iscador P on expression of T-cell receptor-zeta chains of T- and NK cells in 48 patients with early and advanced breast cancer. The finding revealed no consistent change in the expression of zeta-chains on CD4+, CD8+ lymphocytes, and NK cells in patients who underwent mistletoe therapy.

Collecting and combining the results from most of the studies provide a great deal of evidence clearly demonstrating that mistletoe from various origins, particularly European mistletoe, is able to enhance humoral and cellular immune responses in breast cancer patients receiving VAE treatment provided by the subcutaneous route.

## 6. Conclusions and Future Perspectives

This paper summarises the* in vitro*,* in vivo*, and clinical types of evidence available about antibreast cancer efficacy of mistletoe. Currently, mistletoe, especially* V. album*, has attracted special interest due to its vital role as a leading remedy in cancer therapy. The cytotoxic efficacy of mistletoe extracts against cancer cells has been evaluated in numerous evidence based researches as well as* in vitro* and* in vivo* laboratory experiments, and mainly positive antitumor activities have been reported. Laboratory studies indicate that the cytotoxic properties and cell apoptosis inducing activity of mistletoe depend on its biologically active substances which may be different in various geographical regions and host trees. The majority of the included clinical trials suggested a beneficial effect with good evidence with respect to survival, HRQoL, positive remission rate, and reduction of chemotherapy causing side effects for breast cancer patients treated with mistletoe extracts. However, some of the clinical studies do not seem to be reliable enough due to the minor weakness of studies. It is well established that mechanisms underlying the anticancer activity of mistletoe involved apoptosis and immunomodulation/stimulation of proinflammatory cytokines which point to an improved balance of the innate immune system. Moreover, both immunomodulatory and apoptosis/cytotoxic activities of mistletoe may contribute to the positive outcome and clinical benefit in breast cancer patients.

Future preclinical studies are needed to investigate an accurate mechanism of antitumor activity of mistletoe on cancer cell lines. Heterogeneity of tested animal models and the sample size frequently give rise to inconsistent findings. In order to obtain reliable results, dosage standardization is required. Application of standardized and optimized mistletoe may be helpful in prolonging survival time and increasing HRQoL. Subcutaneous application of mistletoe extracts to breast cancer patients might be associated with immunological side effects on the skin. Taking into consideration the subcutaneous administration side effects, oral administration of mistletoe preparations with apparent lack of adverse reactions might be a promising adjuvant alternative therapy for breast cancer patients. Further qualitative clinical trials evaluating the effect of mistletoe on breast cancer patients must address safety parameters, standard dosage, and appropriate endpoint measures. These studies should take into account the sample size, follow-up time, and methodological design limitation of previous mistletoe trials.

## Figures and Tables

**Table 1:** Summary of *in vitro* and *in vivo* studies of mistletoe extracts on breast cancer cells and animal models.

Mistletoe preparations tested	Cell line/animal model used	Main findings	Reference
Triterpenoids and triterpenoids saponin from *Viscum liquidambaricolum *	MCF-7	Positive cytotoxic effect	[50]
Pachypodol and ombuine from *Viscum coloratum*Kom. nakai	MCF-7	Positive cytotoxic effect	[51]
*Viscum album *(unidentified host tree)	MDA-MB 435	Cell viability was reduced significantly.	[52]
Hydroalcoholic extract of * V.albumL.*fromhornbeam tree (*Carpinus betulus*)	MCF7	Positive cytotoxic effect	[53]
Ethanolic extract of* Dendrophthoe falcate *stem (unidentified host tree)	MCF7	Cytotoxic activity increased significantly	[54]
*Phoradendron serotinum *	MCF-7	Positive cytotoxic effect	[56]
Aqueous mistletoe extract with a defined content of bioactive mistletoe lectin	MCF-7	Positive cytotoxic activity, no stimulation of tumor cell proliferation was observed	[63, 64]
Helixor A, Helixor P, and Helixor M	MCF7, MACLMDA-MB231, MACL MDA-MB648	Cytotoxic activity on tumor cells was found	[57]
Iscador M spezial and Iscador QU spezial	MAXF401 NL	Positive anticancer activity	[58]
Iscador M, Iscador Q, and Abnobaviscum Fraxini-2	MFM-223, KPL-1	Proliferation of cells was strongly inhibited	[59]
*VAP-QU, VAP-M, VAP-A, VAP-P, and*purified lectin	MFM-223, MCF-7	Cell growth was reduced	[62]
Iscador M, Iscador Q, Iscador P	MCF7	Induction of apoptosis	[65]
ME-M (Helixor M), and ME-A (Helixor A)	BT474, BALB/c-mouse	Dose dependent cytotoxic effect along with decrease in tumor weight was observed	[60]
Iscador M	MDA-MB-468-HER2	Inhibition of epidermal growth factor induced proliferation of cells	[61]
Phoratoxin from* Phoradendron tomentosum *growing on* Populus fremontii* S. Wats	primary culture of human tumor cell from patients	Significant toxicity on tumor cell was witnessed	[55]
Iscador M *cum argento *	Chemosensitive transplantation tumors model/Rats	No significant antitumor and cytotoxic activities were found	[66]
*Abnoba-Viscum M *	MAXF 449, sc/Nude mice	Cell proliferation was reduced	[67]

**Table 2:** Summary of the clinical trials on efficacy of mistletoe therapy in breast cancer patients.

Product tested	Study design	No. of patients	Main finding	Reference
Iscador M, Iscador Q, and Iscador P	Prospective nonrandomized and randomized matched-pair trial	91 pairs	Patients' self-regulation was enhanced	[91]
Abnoba-Viscum (r)	Retrolective cohort study	689	HRQoL was improved	[92]
Lectin-standardized mistletoe extract (sME)	Cohort study	1248	HRQoL was improved, relapse free interval was prolonged	[93]
*Viscum album L*., Iscador	Comparative, randomized cohort study	1442	Side effects caused by chemotherapy were reduced. Survival was improved	[68]
Helixor A	Randomized multicentric trial	68	Side effects of chemotherapy were alleviated	[69]
Standardized mistletoe extract PS76A2 (Lektinol)	Randomized and double blind trial	272	HRQoL was positively affected	[94]
Standardized mistletoe extract PS76A2 (Lektinol)	Randomized, double blind trial	352	Survival time was prolonged during and after chemotherapy	[70]
Helixor A	Double blind controlled trial	23	No difference in HRQoL was observed in both placebo and Helixor A treated groups	[95]
Iscador	Controlled Randomized and nonrandomized	122 pairs	Survival time was significantly enhanced	[96]
Helixor	Comparative epidemiological cohort study	741	HRQoL was improved; side effects of chemotherapy were reduced	[71]
Iscador M spezial	Randomized controlled trials	65	No effect on HRQOL was observed; adverse reaction to chemotherapy was reduced	[72]
Iscador M spezial	Prospective open 2-armed nonrandomized trial	33	Lower incidence of nausea was observed; HRQoL was improved	[97]
Iscador M spezial	Randomized open label trial	95	Better HRQoL was developed, trend toward neutropenia reduction was observed	[73]
Iscador M	Case study	1	Dose-dependent benefits were obtained in decreasing fatigue	[98]
Abnoba-Viscum Mali	A noninterventional and prospective trial	270	Adverse reactions to chemotherapy were reduced. HRQoL was improved	[74]
Iscador M spezial	Randomized clinical trial	95	HRQoL was improved	[99]
Iscador P, Qut, and M	Multicenter, controlled, and retrolective	3376	Tumor related symptoms decreased significantly and overall survival was prolonged	[100]
Isorel, Iscador Qu, M or P, *abnoba-Viscum Qu or A *	Multi cancer cohort study	4	HRQoL was improved	[101]
Iscador M spezial	Noninterventional follow up trial	57	HRQoL was enhanced; neutropenia was prevented	[75]
Helixor A	prospective randomized open-label	95	HRQoL improved significantly	[102]

**Table 3:** A list of systematic and meta-analysis reviews including controlled randomized, nonrandomized, and matched pair clinical trials on different aspects of HRQoL in breast cancers patients.

Main focus	Main findings	Reference
Effect of mistletoe therapy in reduction of chemotherapy adverse reaction	HRQoL improved in breast cancer patients	[76]
Evaluate efficacy of mistletoe on cancer	Chemotherapy side effects were reduced and HROoL was improved	[77]
Analyze safety and effectiveness of mistletoe in cancer patients	Weak evidence of positive effect on survival of cancer patients was observed; some evidence on the improvement of HRQoL in breast cancer patients was found	[78]
Evaluate the Influence of complementary mistletoe treatment on HRQoL and survival of breast cancer patients	Iscador therapy in addition to chemotherapy might prolong survival and HRQoL	[79]
Effectiveness of mistletoe on cancer patients with respect to HRQoL associated measures	Most of the studies showed improved HRQoL; four studies showed no differences	[81]
Investigate effectiveness of mistletoe on cancer	Most of the trials exhibited beneficial effects; three showed no effect; one indicated negative effect	[82]
Determine impact ofIscador on survival rates of cancer patients	Survival rates were enhanced	[83]
Analyze preclinical and clinical trials on effectiveness of *V.album* on cancer	Positive impacts were observed	[84]
Efficacy of Iscador on HRQoL in cancer patients	Some evidence of positive effect was found	[85]
Retrolective on effectiveness of mistletoe on cancer patients	Positive effects were found; retrolective study design was limited	[86]
Documentation of observational studies and clinical trials with Iscador	Survival rates were enhanced; tumor in breast cancer patients who received Iscador was completely or partially remitted	[87]
